# ANNEXIN A1: Roles in Placenta, Cell Survival, and Nucleus

**DOI:** 10.3390/cells11132057

**Published:** 2022-06-29

**Authors:** Stefanie Oliveira de Sousa, Mayk Ricardo dos Santos, Samuel Cota Teixeira, Eloisa Amália Vieira Ferro, Sonia Maria Oliani

**Affiliations:** 1 Department of Biology, Institute of Biosciences, Humanities and Exact Sciences (Ibilce), São Paulo State University (UNESP), São José do Rio Preto 15054-000, SP, Brazil; stefanie.sousa@unesp.br (S.O.d.S.); mayk.kd@gmail.com (M.R.d.S.); 2 Laboratory of Immunophysiology of Reproduction, Institute of Biomedical Science (ICBIM), Federal University of Uberlândia (UFU), Uberlândia 38400-239, MG, Brazil; samuctx@gmail.com (S.C.T.); eloisa.ferro@ufu.br (E.A.V.F.); 3 Post Graduate Program in Structural and Functional Biology, Escola Paulista de Medicina (UNIFESP-EPM), Federal University of São Paulo, São Paulo 04023-062, SP, Brazil; 4 Advanced Research Center in Medicine (CEPAM), União das Faculdades dos Grandes Lagos (Unilago), São José do Rio Preto 15030-070, SP, Brazil

**Keywords:** human placenta, inflammation, nuclear translocation, gestational diabetes mellitus, peptide Ac_2-26_

## Abstract

The unbiased approaches of the last decade have enabled the collection of new data on the biology of annexin A1 (ANXA1) in a variety of scientific aspects, creating opportunities for new biomarkers and/or therapeutic purposes. ANXA1 is found in the plasma membrane, cytoplasm, and nucleus, being described at low levels in the nuclear and cytoplasmic compartments of placental cells related to gestational diabetic diseases, and its translocation from the cytoplasm to the nucleus has been associated with a response to DNA damage. The approaches presented here open pathways for reflection upon, and intrinsic clarification of, the modulating action of this protein in the response to genetic material damage, as well as its level of expression and cellular localization. The objective of this study is to arouse interest, with an emphasis on the mechanisms of nuclear translocation of ANXA1, which remain underexplored and may be beneficial in new inflammatory therapies.

## 1. Introduction

Annexin A1 (ANXA1), initially described in the late 1970s, was the first characterized member of the annexin superfamily, a group of proteins that attach to the phospholipid membrane in a calcium-dependent manner, and whose anti-inflammatory properties are regulated by glucocorticoids [1,2].

The human ANXA1 gene is located on chromosome 19q24, and encodes a 37-kDa protein. ANXA1 has a central domain (C-terminal), consisting of four repeats of 70 to 80 amino acids, which are highly conserved and responsible for calcium affinity and binding to phospholipids [3,4]. This protein, being a cytosolic protein, when activated by micromolar Ca^2+^, binds to negatively charged phosphatidylserine (PS) to induce membrane cross-linking and promote fusion, essential processes that occur during membrane repair. This is an important function for ANXA1 in general to maintain membrane integrity upon membrane damage [5,6]. Moreover, it has a unique N-terminal domain for each member, containing sites for post-translational processes and the anti-inflammatory protein sequence, which determines its function and biological activity [7].

Classically, ANXA1 is considered a potent endogenous inhibitor of the synthesis of inflammatory mediators, such as eicosanoids, and the activity of the cytosolic phospholipase A2 enzyme (cPLA2), induced by glucocorticoids [8]. Hannon et al. suggested that endogenous ANXA1 expression regulates the expression and/or activity of cPLA2 due to an increase in mRNA and the cPLA2 protein in ANXA1 knockout mice. As this protein is active at intracellular calcium concentrations, ANXA1 may perform important functions in the control of cPLA2 activity [9].

However, since the discovery of ANXA1, it has been implicated in more than just the control of cPLA2 activity, with investigations into its roles in diverse areas, including cardiology, neurology, endocrinology, and oncology [10]. This protein also acts as a regulatory element for several cell types and their associated functions, participating in processes such as the blocking of leukocyte extravasation, induction of apoptosis, modulation of cytokine expression and secretion, activation and regulation of mast cells, proliferation, cell signaling, angiogenesis, migration, tumor invasion, and regulating the blood–brain barrier [11,12,13,14,15,16,17,18].

## 2. ANXA1 and Inflammatory Processes

The protective anti-inflammatory action of ANXA1 has since been demonstrated in several models, including arthritis, heterologous skin transplantation, cancer, eye allergy, heart failure, lung injury, nonalcoholic steatosis, myocardial infarction, and skeletal muscle injury [19,20,21,22,23,24,25,26,27,28,29,30].

Its expression has been observed particularly in cells related to defense processes, such as neutrophils [31,32,33], mast cells [34,35,36], eosinophils [37,38], monocytes [39,40,41], and lymphocytes [8,42,43].

The modulation of ANXA1 anti-inflammatory effects occurs through its binding to the formyl peptide receptor (FPR), a specific class of G protein-coupled transmembrane receptors, and/or through its binding to the phospholipid bilayer of the cell membrane [44]. This functional role is supplied by its peptides derived from the N-terminal region, Ac_2–26_, Ac_2–12_, and Ac_2–6_, which induce the activation of FPR types 1 (FPR1) and 2 (FPR2) [45,46,47].

By understanding the effects of ANXA1 and the cell-specific actions of FPR2, it will be possible to guide the development of new therapies focused on the different physiological responses of the FPR2 agonist to support inflammatory resolution for diseases affecting our society [48].

## 3. ANXA1 in the Placenta

**Figure 1:**
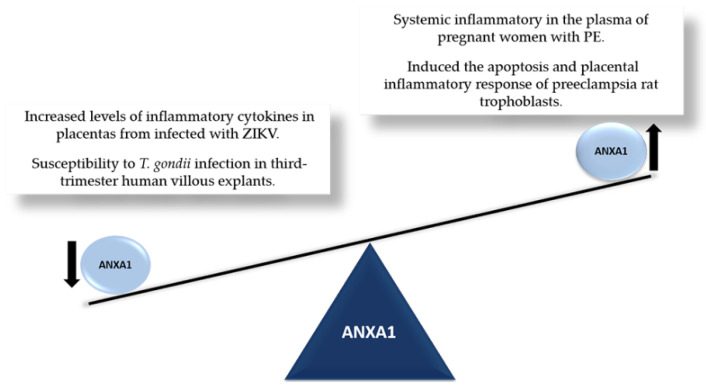
ANXA1 in the placenta. Representation of imbalance in ANXA1 levels in placentas at high-risk in pregnancy: ZIKV [53], *T. gondii* [52] and preeclampsia [59,62].

Recent studies have investigated ANXA1 in different models of inflammation [13,48,49,50,51], including placentas from high-risk pregnancies, such as those associated with *Toxoplasma gondii* [52] and Zika virus (ZIKV) infections [53] (Figure 1).

The placenta acts as a natural barrier between maternal and fetal blood circulation, with endocrine and transport functions. These functions make it not only a crucial regulator of fetal nutrition, gas exchange, and maternal immunological tolerance, but also a target for maternal and fetal metabolic changes associated with pregnancy pathologies [54].

Originally, high levels of ANXA1 expression were described in human uterine tissue, during pregnancy, and in seminal fluid [55], while low levels of this protein were found in the amnion and placenta [56]. More recently, Hebeda et al. suggested that ANXA1 might play a crucial role in the blastocyst implantation phase. Their study shows that this protein controls inflammation, maintains the ideal microenvironment for implantation, interacts with FPR receptors to induce the necessary signaling to activate kinases, and modulates the epithelial cytoskeleton. Furthermore, ANXA1 was found to be related to the dynamic interaction between the uterine epithelium and endothelium, a crucial process for embryo implantation, subsequent decidualization, and, consequently, successful pregnancy [57].

Studies have shown an association between ANXA1 levels and the development of pregnancy-associated diseases such as pre-eclampsia (PE) and gestational diabetes mellitus (GDM). Regarding the functional role of ANXA1 in pregnancy, it was demonstrated that female BALB/c ANXA1 knockout mice presented alterations in the estrogen cycle, an exacerbated inflammatory reaction in the uterine fluid during the implantation phase, and an increase in plasma progesterone at the beginning of pregnancy, resulting in fewer births [58].

Recent investigations involving ANXA1 suggest that the modulation of this protein may be associated with the systemic inflammatory response present in pregnancy-associated pre-eclampsia [59,60]. Behrouz et al. identified that pregnant women with PE had increased levels of autoantibodies against two placental proteins: ANXA1 and the “vitamin D binding protein” in serum. Interestingly, the presence of autoantibodies against ANXA1 was correlated with exacerbated inflammation, typical of pregnancies accompanied by this pathology [61]. Similarly, Perucci et al. reported a significant increase in serum levels of ANXA1 in the plasma of pregnant women with PE, which was associated with a systemic inflammatory phenotype, thus suggesting the deregulation of ANXA1 in the pathogenesis of PE [62]. In an L-NAME-induced PE model in rats, Feng et al. observed the inflammatory response and increased expression of ANXA1 in the placenta, finding that ANXA1 silencing decreased apoptosis, and thus revealing that this protein may contribute to the pathological mechanism of the disease [59].

ANXA1 expression is increased in the placentas of normal pregnancies [18], while lower levels of ANXA1 are present in placentas from high-risk pregnancies, such as in GDM cases that have high levels of inflammatory cytokines [63]. Nonetheless, these authors observed strong immunoreactivity for ANXA1 in the syncytiotrophoblast cytoplasm and nuclei of the syncytial node in placentas from nondiabetic pregnant women, in comparison with placentas from pregnant women with GDM that presented with high levels of inflammatory cytokines. In this context, it has been suggested that ANXA1 plays a role in inflammatory/anti-inflammatory regulatory mechanisms in chorionic villi, which may be crucial in gestational diabetic diseases [63].

Recently, it has been demonstrated that a lower expression of ANXA1 in third-trimester human villous explants is associated with increased susceptibility to *T. gondii* infection. Seeking to corroborate these findings, the researchers also demonstrated that third-trimester villi infected with *T. gondii*, when treated with the synthetic peptide Ac_2–26_, showed an increase in the expression of endogenous ANXA1, resulting in a reduction in the parasitic load [52]. On the other hand, ANXA1 knockout mice infected with the influenza A virus exhibited a survival advantage related to lower virus levels after infection and increased inflammatory cell infiltration [64].

Our research group identified that ANXA1 is highly expressed in the placenta, especially in the syncytiotrophoblast, while there is a decrease in the gene expression of this protein in groups infected with ZIKV [53]. These data suggest that maternal infection with ZIKV is sufficient to develop an inflammatory response in the placenta by increasing the recruitment of cytokines and inflammatory cells, possibly related to ANXA1 modulation.

In addition to mediating the inflammatory process, ANXA1 is involved in important pathophysiological processes, including cell proliferation and differentiation, cancer, and apoptosis; many of these processes relate to the response to DNA damage [65,66,67].

## 4. ANXA1 and Cell Survival

To survive and maintain genome integrity, organisms have DNA repair mechanisms that work effectively to remove lesions [68,69]. Quantitative proteomics studies indicate that ANXA1 may play a role in this DNA damage response [70]. Furthermore, in mammary adenocarcinoma cells (MCF7), it was shown that ANXA1 is related to protein cellular stress by protecting DNA against heat-induced damage [71].

Each type of DNA damage requires a specific set of cellular responses. Depending on the nature of the damage, different mechanisms are needed to repair the genetic material; when the damage exceeds the cell’s ability to repair itself, consequences such as the accumulation of mutations in the genome, or even cell death, may be observed [72,73]. Apoptosis and its associated regulatory mechanisms are crucial physiological events for the maintenance of placental homeostasis, and the imbalance of these processes can, among other consequences, compromise the function of the placenta, and therefore the success of the pregnancy [74].

Choi and collaborators developed an integrative network analysis to identify proteins that respond to the ATM inhibitor (a protein with a central role in the DNA damage signaling cascade) and physical interactions with DNA repair proteins. Interestingly, the analysis identified 53BP1 and ANXA1 as strong candidates. Complementing these results, the authors demonstrated that cell lines that do not express the ANXA1 protein are more sensitive to ionizing radiation [75].

Apoptosis is a physiological process of cell death in which cells undergo structural changes and are removed from the body without triggering an inflammatory response. The first indication of the involvement of ANXA1 in apoptosis was reported by McKanna, who showed that the expression of ANXA1 increased in alveolar cells of the mammary ducts undergoing apoptosis in post-lactational regression [76]. Subsequently, Sakamoto et al. reported that exogenous ANXA1 facilitated hydrogen peroxide-induced apoptosis in rat thymocytes [77]. Further evidence suggests that ANXA1 can mediate the proapoptotic effects of glucocorticoids in some cells, activating caspase-3 and acting on calcium fluxes [78,79].

## 5. ANXA1 in the Nucleus

ANXA1 has been found in the plasma membrane, cytoplasm, and nucleus [80,81]. Several studies have focused on the nuclear localization of ANXA1 (Table 1), and its translocation from the cytoplasm to the nucleus has been reported as a response to DNA damage, proliferative stimuli, and phosphorylation [82]. In addition, when overexpressed, intra- and/or extracellular ANXA1 translocated to the nucleus during apoptosis [83].

Studies indicate that ANXA1 nuclear translocation may be associated with cancer progression through the regulation of transcription factors and miRNAs [84], and the induction of apoptosis through the regulation of transcription factors such as p53 and p65 [85]. Recently, Luo et al. reported that ANXA1 determines the fate of retinal ganglion cells in a murine glaucoma model, and that its nuclear translocation induces apoptosis in these cells [86]. In addition, other works have shown that, when translocated to the nucleus, ANXA1 participates in neuronal apoptosis after cerebral ischemia [85,87]. In particular, the protein was found to act as a cofactor, binding to p53 in the nucleus and positively regulating its transcriptional activity, leading to the expression of the proapoptotic BID gene. Blocking ANXA1 nuclear translocation via a specific β-importin inhibitor reduced BID expression and inhibited the activation of the caspase-3 apoptotic pathway, attenuating neuronal apoptosis after ischemic stroke [87,88].

Although ANXA1 does not contain a classical nuclear localization signal, it has been observed that in the ANXA1 repeat domain III, amino acid residues from R228 to F237 function as a single nuclear translocation signal (NTS), and are required for the nuclear translocation of ANXA1 [82]. A recent study by Xia et al. found that the intracerebroventricular injection of the recombinant adenovirus vector S100A11 protects cells by preventing cerebral ischemia-induced neuronal cell apoptosis. Through the NTS, the adenovirus interacted directly with ANXA1, markedly decreasing its nuclear translocation [89].

Rhee and collaborators, aiming to identify whether the protein ANXA1 was related to cellular stress, observed that its gene expression levels increased in cells treated under stress conditions. Furthermore, in response, ANXA1 is translocated from the cytoplasm to the nucleus and perinuclear region. Its role in resolving stress-induced transcriptional activation was investigated, and the associated alteration was significantly larger than in cells maintained under different conditions [90].

The presence of ANXA1 in the nucleus has also been suggested as a significant predictor of survival in oral and esophageal squamous cell carcinomas. It was observed that ANXA1 expression, although decreased in the cytosol and membranes, was increased in the nuclei of esophageal cancer cells. Furthermore, patients with low nuclear ANXA1 expression had better prognoses than those with high protein expression [67,91]. Similar studies showed that ANXA1 is expressed in both gastric adenocarcinoma and normal tissues. In gastric adenocarcinoma tissues, ANXA1 is expressed in both the cytoplasm and the nucleus, and its nuclear location correlates with the advanced stage of the disease and peritoneal dissemination [92]. Moreover, it was demonstrated in L5178Y tk^+/−^ mouse lymphoma cells treated with DNA-damaging agents that the quantity of nuclear ANXA1 increased while cytoplasmic ANXA1 levels decreased, suggesting that nuclear translocation of this protein occurs in response to the signaling of damaged DNA [93].

Considered in combination, these findings from the literature indicate that, in addition to its level of expression, the subcellular localization and translocation of ANXA1 may play an important role in several pathologies.

**Table 1:** ANXA1 in the nucleus.

ANXA1 in Nucleus		
Model	Functions	Ref.
Ischemia-reperfusion injury	Nuclear translocation induced neuron and retinal ganglion cell apoptosis	[85,86,87]
Ischemic stroke	Nuclear translocation reduced BID expression and inhibited the activation of the caspase-3 apoptotic pathway, attenuating neuronal apoptosis	[88]
Cellular stress	Gene expression levels increased, and translocation of annexin I from the cytoplasm to the nucleus initiated, in cells treated under stress conditions	[90]
Oral and esophageal squamous cell carcinoma	Patients with low nuclear ANXA1 expression had better prognoses than those with high protein expression	[67,91]
Gastric adenocarcinoma	Nuclear location correlated with the advanced stage of the disease and peritoneal dissemination	[92]

## 6. Perspective and Conclusions

Since its discovery as an anti-phospholipase protein, ANXA1 has been found to exhibit a wide range of anti-inflammatory properties. However, further research is needed to define the processes and factors that influence its nuclear translocation, with the aim of identifying the mechanisms by which this protein performs its functions in the nucleus.

Although this discussion has focused on the placenta, this review provides novel insights into how ANXA1 regulates the body’s pathophysiological processes, predominantly in relation to its nuclear action. This aspect will be particularly important for further investigation into the role of ANXA1 in the nucleus, and the development of new inflammatory therapies based on the understanding and targeting of this protein.

## Data Availability

Not applicable.
